# Supplementary material for: Oligomerization state of the functional bacterial twin-arginine translocation (Tat) receptor complex
Source: Commun Biol. 2022 Sep 19;5:988. doi: 10.1038/s42003-022-03952-2 (PMC9485244; doi:10.1038/s42003-022-03952-2)
Supplement: Supplementary file 2 — Supplemental Information [file 42003_2022_3952_MOESM2_ESM.pdf]

SUPPLEMENTARY INFORMATION

for

Oligomerization state of the functional bacterial twin arginine translocation (Tat) receptor complex

Ankith Sharma, Rajdeep Chowdhury and Siegfried M. Musser*

Department of Molecular and Cellular Medicine, Texas A&M University, School of Medicine, 1114 TAMU, College Station, TX 77843, USA

*Corresponding author, e-mail: smusser@tamu.edu

SUPPLEMENTARY TABLE 1. Summary of best fit values obtained for photobleaching histograms assuming different oligomerization states of the Tat receptor complex[1]

TatB^{mNeon}:

	Poisson Mean	FDE	RMSD		Predicted Empty IMVs (%)
			Steps 2-8	Steps 1-8	
**Monomers**	3.9	0.95	0.035	0.038	2.4
	3.9	0.86	0.035	0.034	3.5
	3.5	0.95	0.032	0.030	3.6
	3.3	0.98	0.032	0.031	3.9
**Dimers**	3.9	0.47	0.035	0.048	6.2
	3.6	0.46	0.031	0.040	7.9
	2.9	0.56	0.024	0.033	9.8
	3.8	0.41	0.030	0.039	8.5
**Trimers**	2.9	0.42	0.037	0.053	10.2
	2.5	0.43	0.031	0.044	13.4
	2.7	0.39	0.023	0.036	12.8
	3.7	0.33	0.031	0.039	7.8
**Tetramers**	1.1	0.70	0.010	0.010	35.6
	1.0	0.65	0.013	0.012	38.8
	1.1	0.60	0.013	0.012	35.5
	0.9	0.64	0.008	0.008	42.2
**Pentamers**	0.9	0.59	0.013	0.015	43.2
	0.9	0.53	0.019	0.017	42.9
	1.1	0.48	0.015	0.016	36.1
	0.8	0.52	0.017	0.014	46.9
**Hexamers**	0.8	0.50	0.018	0.021	47.5
	0.9	0.43	0.021	0.023	43.3
	1.0	0.41	0.016	0.020	39.8
	0.8	0.43	0.020	0.019	47.3
**Heptamers**	0.8	0.43	0.021	0.025	47.7
	0.9	0.38	0.022	0.024	43.6
	1.0	0.35	0.017	0.021	40.1
	0.8	0.37	0.022	0.021	47.6
**Octamers**	0.8	0.37	0.023	0.029	47.9
	0.8	0.33	0.024	0.026	47.8
	1.0	0.30	0.017	0.024	40.4
	0.7	0.33	0.023	0.023	52.2

TatC^{mNeon}:

	Poisson Mean	FDE	RMSD		Predicted Empty IMVs (%)
			Steps 2-8	Steps 1-8	
**Monomers**	3.8	0.74	0.024	0.060	6.2
	3.9	0.82	0.033	0.039	4.1
	3.3	0.98	0.038	0.037	3.8
	3.4	0.82	0.028	0.053	6.2
**Dimers**	3.4	0.39	0.027	0.074	11.9
	3.1	0.50	0.027	0.049	9.9
	2.9	0.55	0.022	0.039	9.3
	3.6	0.37	0.030	0.068	11.5
**Trimers**	2.4	0.35	0.031	0.080	17.7
	2.5	0.40	0.027	0.052	14.4
	1.5	0.68	0.019	0.019	24.4
	3.2	0.27	0.031	0.073	14.3
**Tetramers**	0.7	0.61	0.009	0.030	51.1
	1.0	0.62	0.014	0.016	38.3
	1.2	0.58	0.013	0.014	32.6
	0.6	0.62	0.015	0.022	56.0
**Pentamers**	0.59	0.49	0.012	0.037	57.1
	0.9	0.50	0.016	0.022	43.0
	1.2	0.45	0.016	0.023	33.2
	0.5	0.50	0.017	0.030	61.9
**Hexamers**	0.5	0.42	0.015	0.040	62.2
	0.9	0.41	0.018	0.020	43.4
	1.2	0.37	0.018	0.027	33.8
	0.5	0.41	0.019	0.038	62.3
**Heptamers**	0.4	0.37	0.017	0.042	68.4
	0.8	0.36	0.019	0.030	47.6
	1.2	0.31	0.018	0.031	34.2
	0.5	0.35	0.021	0.042	62.6
**Octamers**	0.37	0.32	0.018	0.046	70.5
	0.8	0.31	0.020	0.034	47.9
	1.2	0.27	0.019	0.032	34.5
	0.5	0.30	0.022	0.047	62.8

[1]The first two rows (*black*) for each oligomer size are the values obtained using manual step counting for two different preparations of IMVs containing TatAB^{mNeon}C or TatABC^{mNeon} (histograms with tetramer fits in **Supplementary Fig. 11).** The third rows (*red*) are the results obtained by analyzing the data used in the second row with the PDD function (histograms with tetramer fits in **Supplementary Fig. 12)**. The fourth rows (*blue*) are the '2 h chase' datasets analyzed using the PDD function (histograms with monomer to octamer fits in **Figs. 6** and **8** and **Supplementary Fig. 10)**.

H6-mNeon
(plasmid pH6-mNeon; Addgene #178460):
MGHHHHHHTSMVSKGEEDNMASLPATHELHIFGSINGVDFDMVGQGTGNPNDGYEELNLKSTKGDLQFSPWILVPHI
GYGFHQYLPYPDGMSPFQAAMVDGSGYQVHRTMQFEDGASLTVNYRYTYEGSHIKGEAQVKGTGFPADGPVMTNSLT
AADWCRSKKTYPNDKTIISTFKWSYTTGNGKRYRSTARTTYTFAKPMAANYLKNQPMYVFRKTELKHSKTELNFKEW
QKAFTDVMGMDELYK

H6-mNeon-C
(plasmid pH6-mNeonC; Addgene #178461):
MGHHHHHHTSMVSKGEEDNMASLPATHELHIFGSINGVDFDMVGQGTGNPNDGYEELNLKSTKGDLQFSPWILVPHI
GYGFHQYLPYPDGMSPFQAAMVDGSGYQVHRTMQFEDGASLTVNYRYTYEGSHIKGEAQVKGTGFPADGPVMTNSLT
AADWCRSKKTYPNDKTIISTFKWSYTTGNGKRYRSTARTTYTFAKPMAANYLKNQPMYVFRKTELKHSKTELNFKEW
QKAFTDVMGMDELYKC

H6-2xmNeon
(plasmid pH6-2xmNeon; Addgene #178462):
MGHHHHHHTSGTMVSKGEEDNMASLPATHELHIFGSINGVDFDMVGQGTGNPNDGYEELNLKSTKGDLQFSPWILVP
HIGYGFHQYLPYPDGMSPFQAAMVDGSGYQVHRTMQFEDGASLTVNYRYTYEGSHIKGEAQVKGTGFPADGPVMTNS
LTAADWCRSKKTYPNDKTIISTFKWSYTTGNGKRYRSTARTTYTFAKPMAANYLKNQPMYVFRKTELKHSKTELNFK
EWQKAFTDVMGMDELYKQLGSGSTSMVSKGEEDNMASLPATHELHIFGSINGVDFDMVGQGTGNPNDGYEELNLKST
KGDLQFSPWILVPHIGYGFHQYLPYPDGMSPFQAAMVDGSGYQVHRTMQFEDGASLTVNYRYTYEGSHIKGEAQVKG
TGFPADGPVMTNSLTAADWCRSKKTYPNDKTIISTFKWSYTTGNGKRYRSTARTTYTFAKPMAANYLKNQPMYVFRK
TELKHSKTELNFKEWQKAFTDVMGMDELYK

H6-2xmNeon-C
(plasmid pH6-2xmNeonC); Addgene #178463):
MGHHHHHHTSGTMVSKGEEDNMASLPATHELHIFGSINGVDFDMVGQGTGNPNDGYEELNLKSTKGDLQFSPWILVP
HIGYGFHQYLPYPDGMSPFQAAMVDGSGYQVHRTMQFEDGASLTVNYRYTYEGSHIKGEAQVKGTGFPADGPVMTNS
LTAADWCRSKKTYPNDKTIISTFKWSYTTGNGKRYRSTARTTYTFAKPMAANYLKNQPMYVFRKTELKHSKTELNFK
EWQKAFTDVMGMDELYKQLGSGSTSMVSKGEEDNMASLPATHELHIFGSINGVDFDMVGQGTGNPNDGYEELNLKST
KGDLQFSPWILVPHIGYGFHQYLPYPDGMSPFQAAMVDGSGYQVHRTMQFEDGASLTVNYRYTYEGSHIKGEAQVKG
TGFPADGPVMTNSLTAADWCRSKKTYPNDKTIISTFKWSYTTGNGKRYRSTARTTYTFAKPMAANYLKNQPMYVFRK
TELKHSKTELNFKEWQKAFTDVMGMDELYKC

TatB-mNeon
(encoded in plasmid pTatAB^{mNeon}C; Addgene #178464):
VFDIGFSELLLVFIIGLVVLGPQRLPVAVKTVAGWIRALRSLATTVQNELTQELKLQEFQDSLKKVEKASLTNLTPE
LKASMDELRQAAESMKRSYVANDPEKASDEAHTIHNPVVKDNEAAHEGVTPAAAQTQASSPEQKPETTPEPVVKPAA
DAEPKTAAPSPSSSDKPYTRVPMVSKGEEDNMASLPATHELHIFGSINGVDFDMVGQGTGNPNDGYEELNLKSTKGD
LQFSPWILVPHIGYGFHQYLPYPDGMSPFQAAMVDGSGYQVHRTMQFEDGASLTVNYRYTYEGSHIKGEAQVKGTGF
PADGPVMTNSLTAADWCRSKKTYPNDKTIISTFKWSYTTGNGKRYRSTARTTYTFAKPMAANYLKNQPMYVFRKTEL
KHSKTELNFKEWQKAFTDVMGMDELYK

TatC-mNeon
(encoded in plasmid pTatABC^{mNeon}; Addgene #178465):
MSVEDTQPLITHLIELRKRLLNCIIAVIVIFLCLVYFANDIYHLVSAPLIKQLPQGSTMIATDVASPFFTPIKLTFM
VSLILSAPVILYQVWAFIAPALYKHERRLVVPLLVSSSLLFYIGMAFAYFVVFPLAFGFLANTAPEGVQVSTDIASY
LSFVMALFMAFGVSFEVPVAIVLLCWMGITSPEDLRKKRPYVLVGAFVVGMLLTPPDVFSQTLLAIPMYCLFEIGVF
FSRFYVGKGRNREEENDAEAESEKTEEVDMVSKGEEDNMASLPATHELHIFGSINGVDFDMVGQGTGNPNDGYEELN
LKSTKGDLQFSPWILVPHIGYGFHQYLPYPDGMSPFQAAMVDGSGYQVHRTMQFEDGASLTVNYRYTYEGSHIKGEA
QVKGTGFPADGPVMTNSLTAADWCRSKKTYPNDKTIISTFKWSYTTGNGKRYRSTARTTYTFAKPMAANYLKNQPMY
VFRKTELKHSKTELNFKEWQKAFTDVMGMDELYK

Supplementary Figure 1. Protein sequences for the mNeon proteins used in this study. The mNeon sequences are indicated in *green*, additions/linkers are in *red*, 6xHis tags are in *blue* and the TatB and TatC sequences are *underlined*. The RVP sequence in the linker of TatB-mNeon reflects an introduced KpnI restriction site.

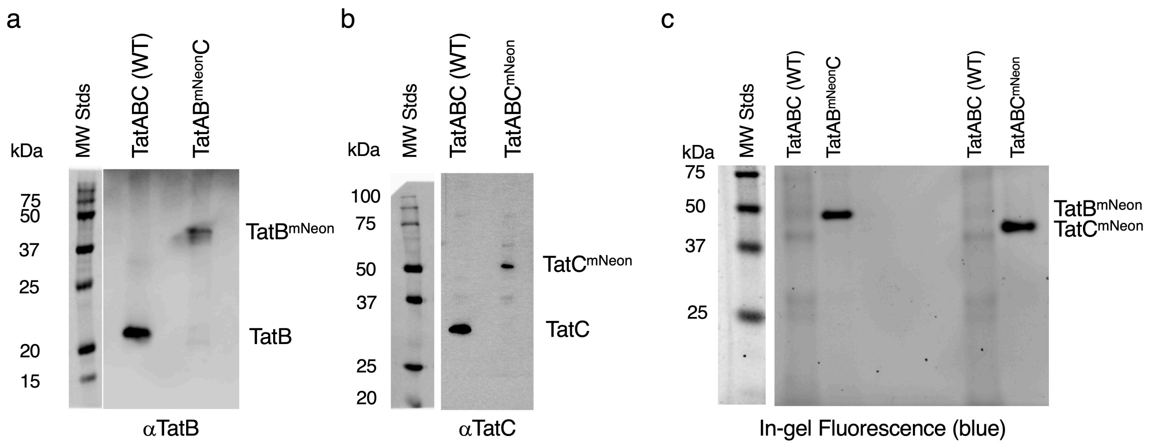

Supplementary Figure 2. Stability of TatB^{mNeon} and TatC^{mNeon} Fusions. a,b, Western blots of IMVs containing TatAB^{mNeon}C or TatABC^{mNeon} were probed with anti-TatB (1:4000) (**a**) and anti-TatC (1:1250) (**b**) antibodies. The shift in the TatB^{mNeon}- and TatC^{mNeon}-containing lanes relative to the bands in the wild-type TatABC lanes arises from the mNeon tag (~25 kDa). No free TatB or TatC was observed for the mNeon-tagged proteins, indicating that the mNeon fusion proteins were not subject to proteolysis. **c,** In-gel fluorescence of IMVs containing TatAB^{mNeon}C or TatABC^{mNeon}. Fluorescence bands ("blue" indicates excitation at 460-490 nm) were detected at a molecular weight consistent with TatB^{mNeon} and TatC^{mNeon} (compare with **a**). The absence of free mNeon confirms that the mNeon fusion proteins were not subject to proteolysis. The slightly different apparent molecular weights of the Tat fusion proteins with mNeon is ascribed to the 4.5 M urea that was added to the sample denaturation buffer in (**a**) and (**b**), but not for the samples in (**c**).

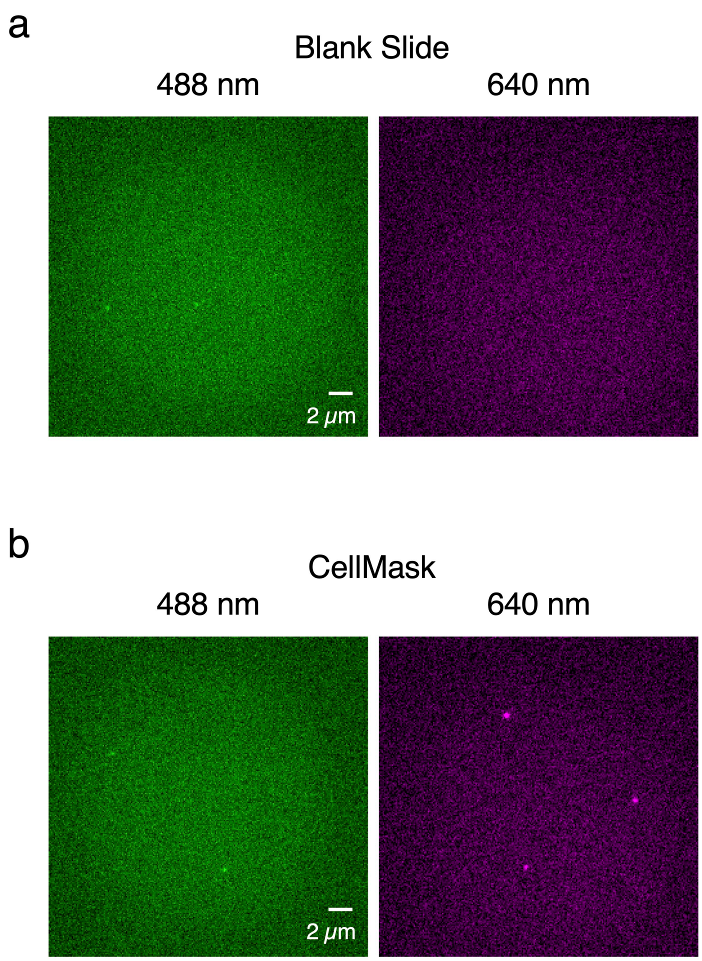

Supplementary Figure 3. Fluorescence Background. a, Fluorescence observed for a plasma-cleaned coverslip covered with Dilution Buffer. An average of 1.9 spots/field were observed with λ_{ex} = 488 nm that photobleached in a single step. Zero spots/field were observed at λ_{ex} = 640 nm. **b**, Fluorescence observed for a plasma-cleaned coverslip treated with 0.1X CellMask dye. The CellMask dye was added in Dilution Buffer, incubated for 15 min at RT, and then washed away. An average of 1.9 and 2 spots/field were observed at λ_{ex} = 488 nm and λ_{ex} = 640 nm, respectively.

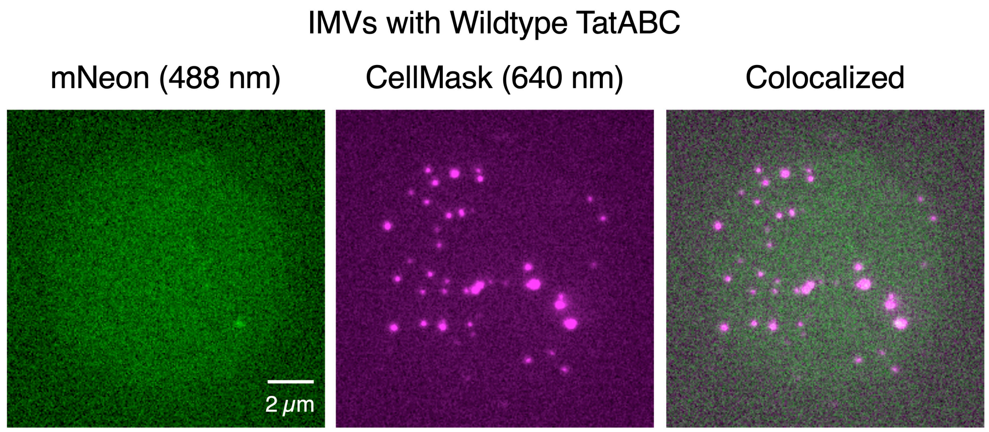

Supplementary Figure 4. Imaging of IMVs Containing Wildtype TatABC Labeled with the CellMask Dye. Approximately 2.7% of CellMask-positive entities were visible with 488 nm excitation. As these spots almost invariably photobleached in a single step, they contaminated the single step histograms in the photobleaching analysis. The data in **Figs. 8** and **Supplementary Figs. 10-12** were corrected based on this background contamination.

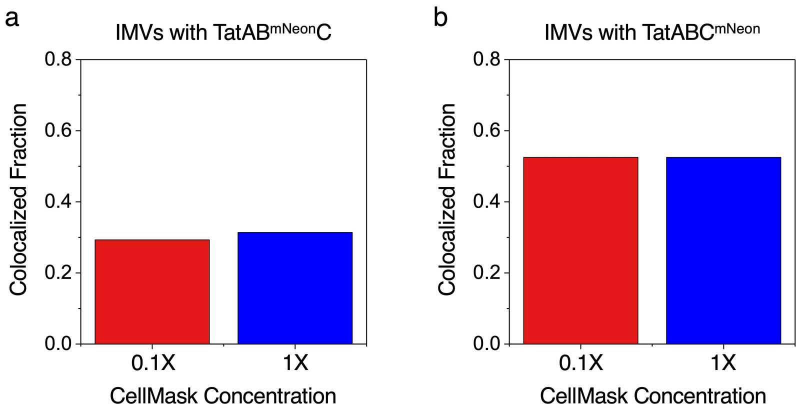

Supplementary Figure 5. CellMask Labeling Efficiency. a,b, IMVs containing TatAB^{mNeon}C (**a**) or TatABC^{mNeon} (**b**) were incubated with two different concentrations of CellMask (15 min at room temperature). The number of mNeon spots that colocalized with the CellMask dye did not substantially change upon increasing the concentration of CellMask by 10-fold. The lower (0.1X) concentration was used for all photobleaching experiments. *N* = number of mNeon-positive spots observed: (**a**) *N* = 480 (0.1X) and 475 (1X); (**b**) *N* = 370 (0.1X) and 130 (1X).

IMVs with TatABC^{mNeon} Generated after a 2 h Chase

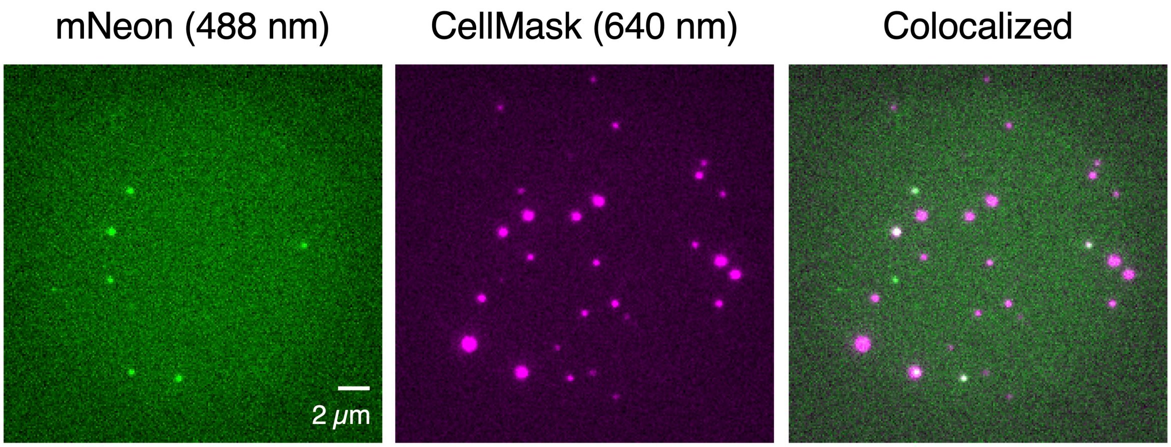

Supplementary Figure 6. Single Particle Imaging of IMVs with TatABC^{mNeon} Obtained after a 2 h Chase. The IMVs imaged here were obtained from cells in which TatABC^{mNeon} was overproduced for 1 h with 0.7% arabinose and then chased without arabinose for 2 h to increase the integration of Tat proteins in the membrane. IMVs prepared in this manner yielded a ~50%-80% colocalization of mNeon with CellMask, a substantial increase from the ~30-50% colocalization without the 2 h chase (compare with **Fig. 3a**). The lower number of spots observed (on average) in the mNeon channel is consistent with a dilution of the Tat receptor complexes in the cytoplasmic membrane due to additional cell divisions.

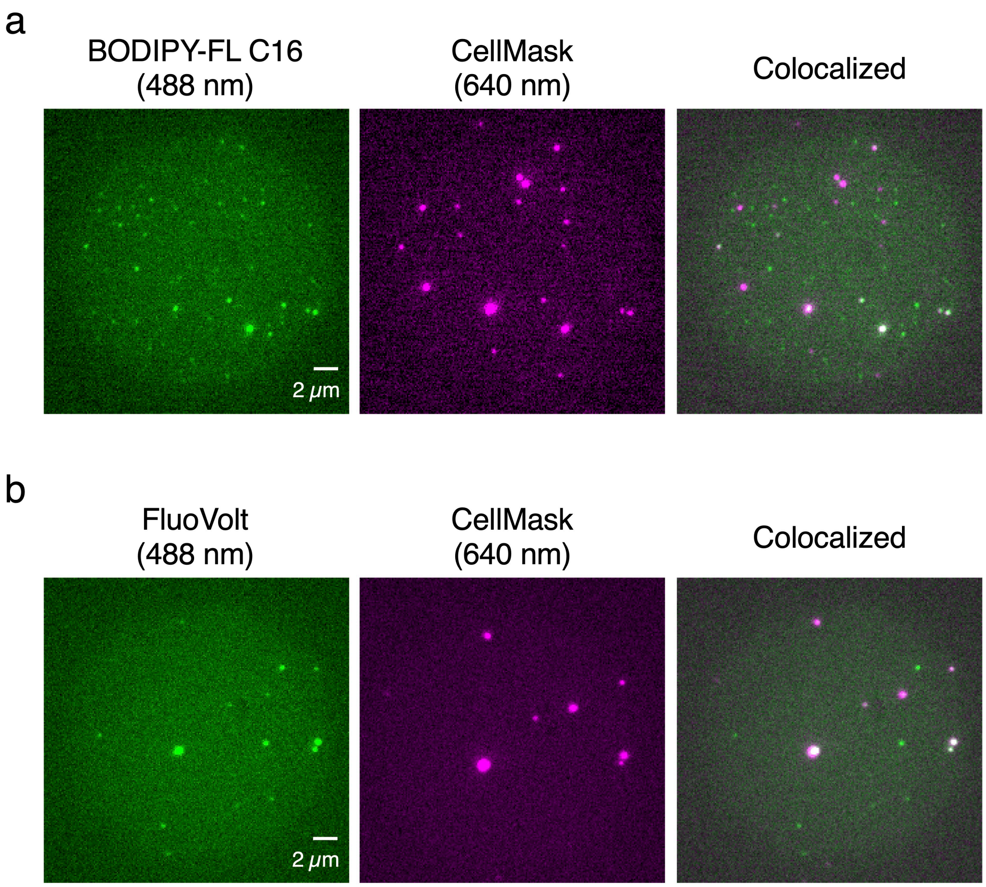

Supplementary Figure 7. IMV Labeling with Different Membrane Dyes. IMVs containing TatABC^{mNeon} were co-incubated with 100 pM BODIPY FL C16 and CellMask (0.1X) (**a**) or FluoVolt (2X) and CellMask (0.1X) (**b**). The quantification of the colocalization is summarized in **Fig. 3c**.

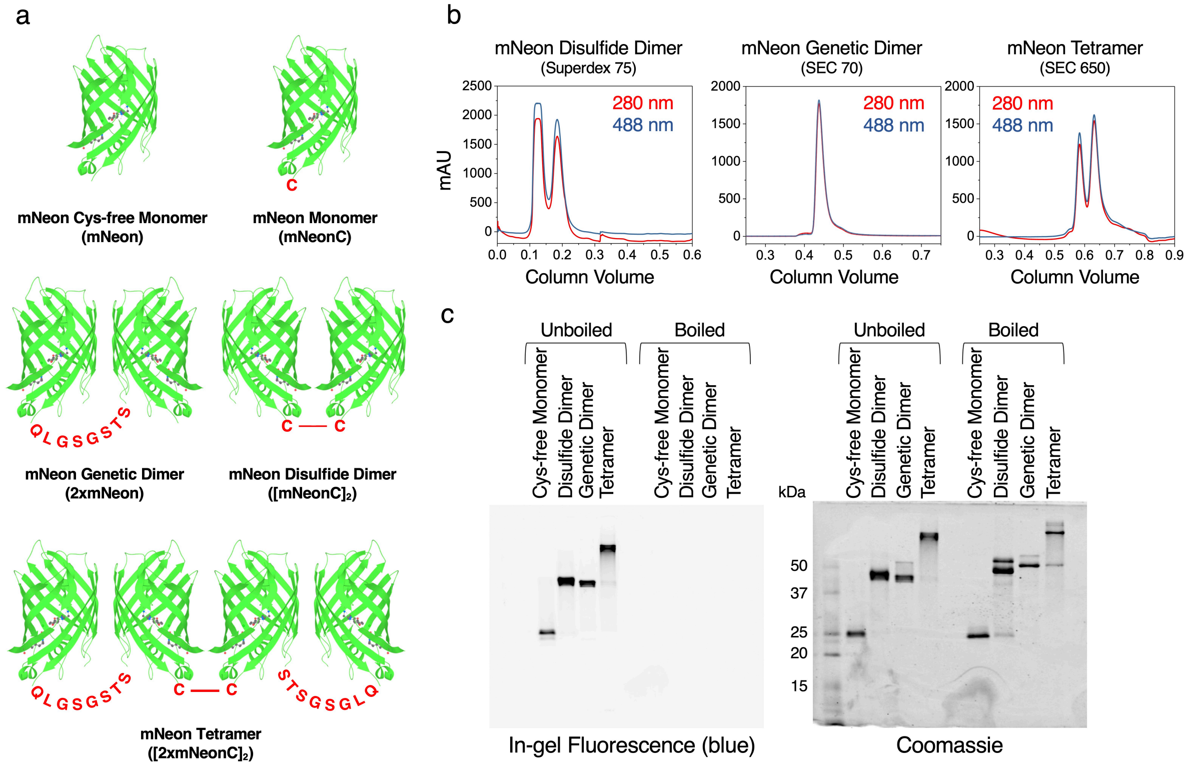

Supplementary Figure 8. Purification of mNeon Standards. a, The mNeon standards. The N-terminal 6xHis-tags are identified in **Supplementary Fig. 1**. The disulfide dimer was generated by a disulfide bond between mNeon monomers with a C-terminal cysteine. The tetramer was generated by a disulfide bond between two genetic dimers with a C-terminal cysteine. **b,** Gel filtration chromatograms. The columns used are indicated. **c,** In-gel fluorescence and Coomassie stained SDS-PAGE gel images of purified mNeon standards. The monomer was purified by Ni-NTA alone; dimers and the tetramer were further purified by gel exclusion chromatography (**b**). Boiling destroys the mNeon fluorescence. The difference of ~25 kDa between monomer and dimer corresponds to the molecular weight of mNeon. "Blue" indicates excitation at 460-490 nm.

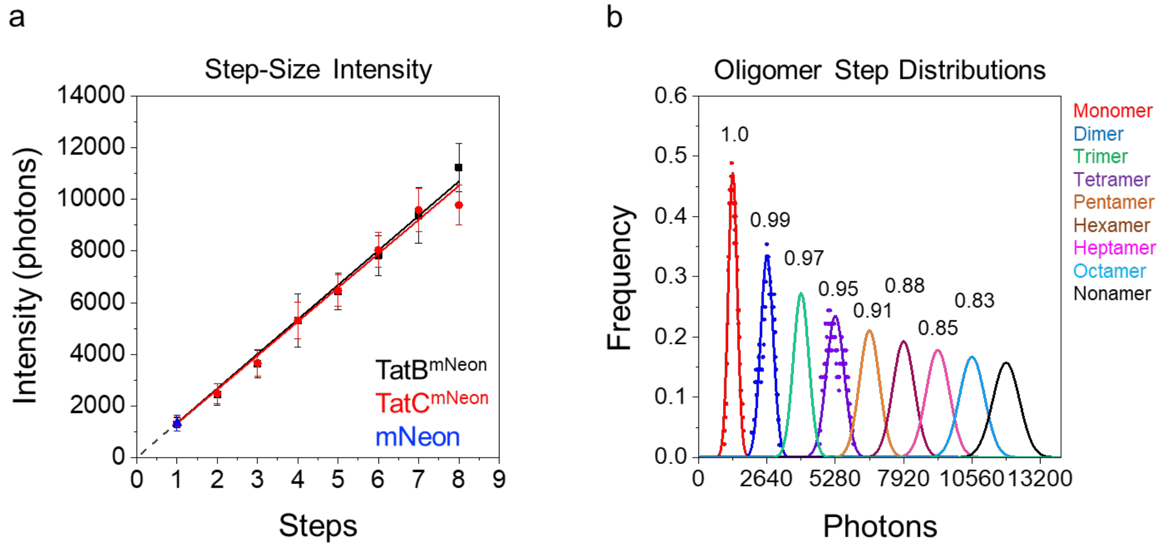

Supplementary Figure 9. Manual and PDD Step Analysis. **a,** The linearity of mean photobleaching step-size intensities for IMVs containing TatAB^{mNeon}C or TatABC^{mNeon} (1 h overproduction). Steps were identified manually. For comparison, the mean intensity obtained for Cys-free mNeon monomers is shown. The error bars (SDs) reveal a significant variation in total intensities, though the steps themselves were generally visually identifiable (**Fig. 4**). Data were fit with a line passing through 0, yielding 1340±20 and 1300±20 photons/step (mean ± standard error) for TatB^{mNeon} and TatC^{mNeon}, respectively. N = 7-112 independent spot intensities (listed in Supplementary Data). **b,** PDD analysis of photobleaching intensities. Rolling bin histograms (200 nm bins, center stepping by 20 nm) of maximal intensities from photobleaching traces of mNeon (monomers), $[\text{mNeonC}]_2$ (disulfide dimers), and $[\text{2xmNeonC}]_2$ (tetramers) were determined (*red*, *blue*, and *purple*, respectively). The monomer histogram was fit with the Gaussian-like expression $y = A*\exp[-(x - 1320)^2/(2\sigma^2)]$, where 1320 is the mean photons/step as determined in (**a**), which yielded $A = 0.472$ and $\sigma = 172$. Assuming the rules for summing independent normally distributed variables, the distribution of intensities for higher order oligomers is then given by $y = (A/n^{1/2})*\exp[-(x - n*1320)^2/(2n\sigma^2)]$, where n is the oligomer size. As this expression fit the dimer and tetramer data very well, the predicted distributions for other oligomer sizes up to nonamer are also shown. The crossing points between neighboring step intensity distributions were taken as the edges of the regions for a given step size. Steps for monomers and low order oligomers could be determined with high confidence, yet confidence decreased for higher order oligomers. The number above each curve is the probability that the intensity determined is assigned the correct step number (i.e., between the crossing points with the neighboring curves). These values substantially overestimate the true errors, as incorrectly assigned intensities will be largely compensated by intensities from the neighboring step bins (which will be dictated by the ground truth of the underlying step histogram).

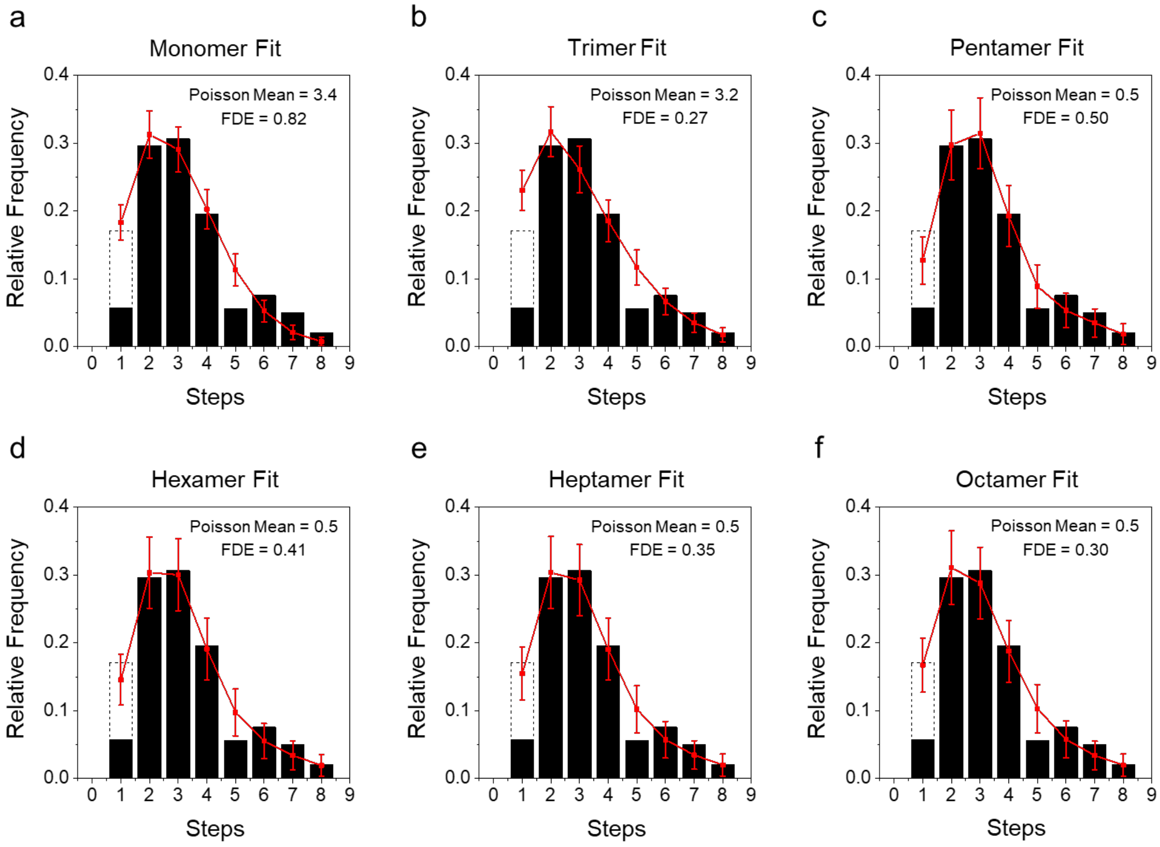

Supplementary Figure 10. Alternate Fits Assuming Different Oligomeric Sizes for the Tat Receptor Complex. While a tetramer model for TatC in the Tat receptor complex yielded the best fit to the '2 h chase' data in **Fig. 8b**, the fits for alternate oligomeric models are shown here (*N* = 240). The monomer fit is shown in **Fig. 6b**. Fit parameters are summarized in **Figs. 8c,d,e** and **Supplementary Table 1**. Error bars are standard deviations (SDs) obtained from 5000 simulated distributions of *N* values each, where *N* is the number of experimental measurements.

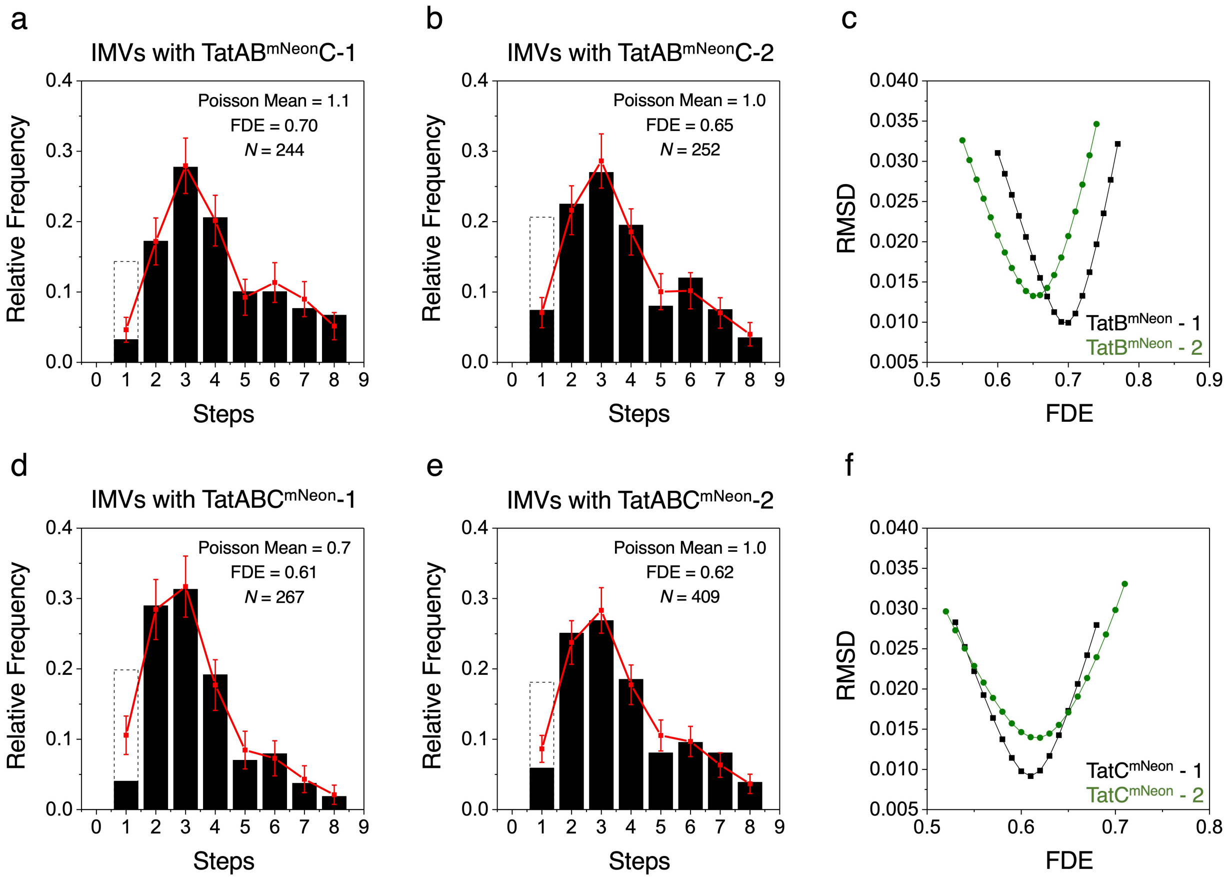

Supplementary Figure 11. Photobleaching Step Histograms for IMVs Containing TatAB^{mNeon}C or TatABC^{mNeon} Obtained after 1 h Overproduction. Two independent (biological duplicate) datasets for IMVs containing TatAB^{mNeon}C or TatABC^{mNeon} showing the best fits assuming a tetramer model (compare with **Fig. 8**). Parameters for the different oligomeric fits are reported in **Fig. 8c,d,e** and **Supplementary Table 1** (in all cases, the data are in *black*). Error bars are standard deviations (SDs) obtained from 5000 simulated distributions of *N* values each, where *N* is the number of experimental measurements from a single IMV preparation, as indicated.

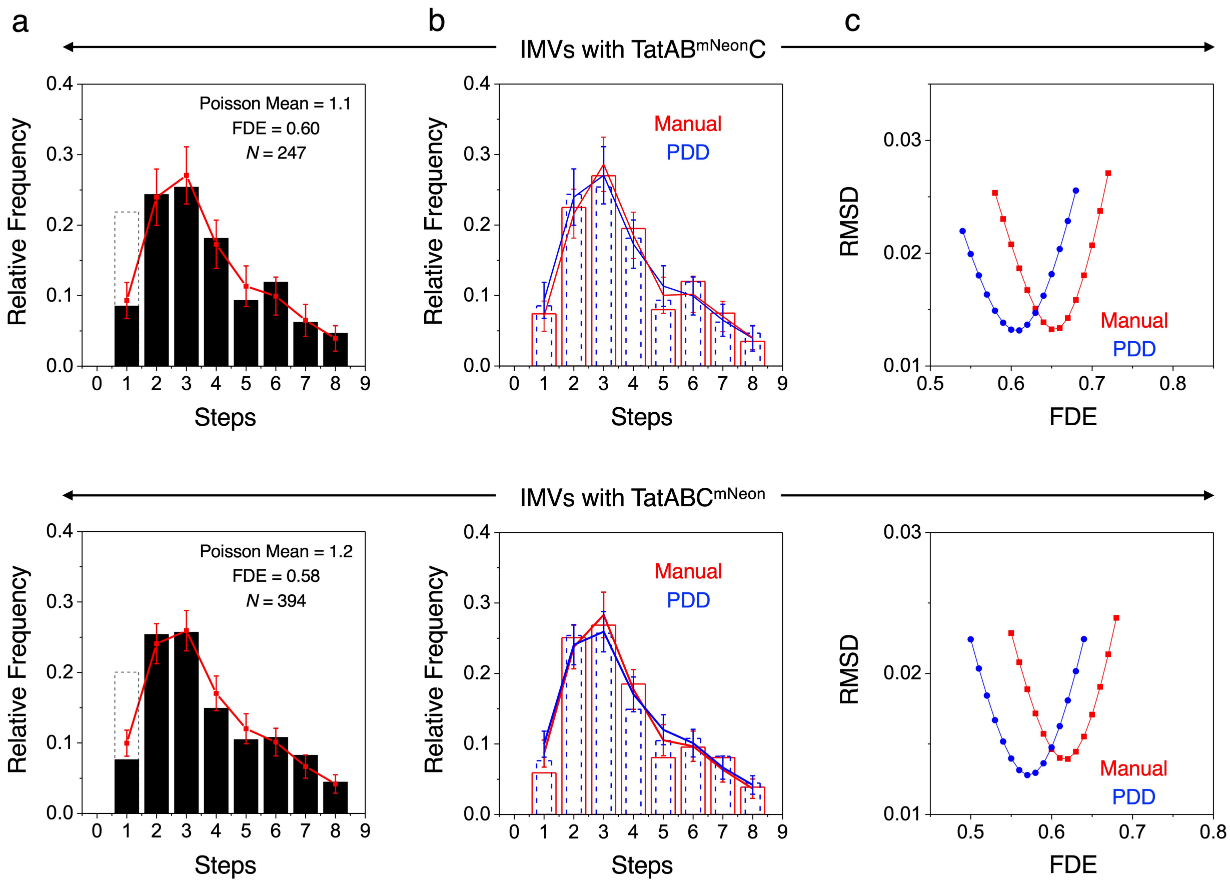

Supplementary Figure 12. Comparison of the Manual and PDD Step-Counting Methods. a, Results of PDD analysis. The datasets used to obtain the histograms in **Fig. 11b,e** were re-analyzed with the PDD algorithm. The best fits (shown here) assume a tetramer model. Parameters for the different oligomeric fits are reported in **Fig. 8c,d,e** and **Supplementary Table 1** (in all cases, the data are in *red*). **b,** Similarity between the results obtained by the manual and PDD step-counting methods. The results from **Supplementary Fig. 11b,e** and **Supplementary Fig. 12a** are overlaid. **c,** FDE optimization for the data in (**b**). The Poisson means were 1.0 for the manual analyses, and 1.1 and 1.2 for the $TatB^{mNeon}$ and $TatC^{mNeon}$ PDD analyses, respectively. Error bars are standard deviations (SDs) obtained from 5000 simulated distributions of *N* values each, where *N* is the number of experimental measurements from a single IMV preparation, as indicated.

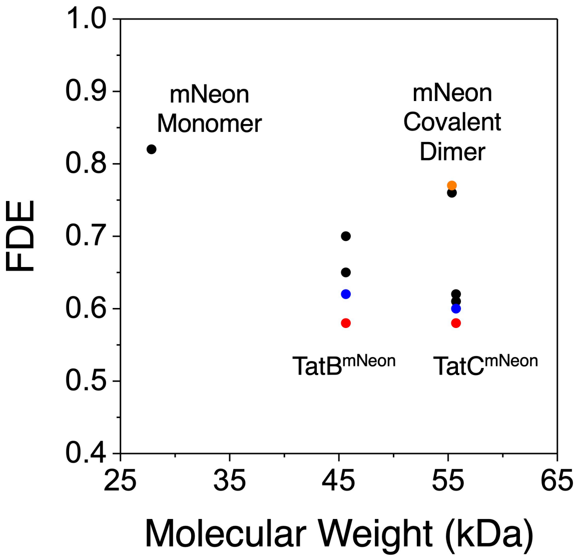

Supplementary Figure 13. FDE vs Molecular Weight for Different mNeon Constructs. Summary of FDE values for mNeon determined in this study. For TatB^{mNeon} and TatC^{mNeon}, the color coding of **Supplementary Table 1** was used. In the mNeon covalent dimer cluster, the *orange* dot represents the mNeon tetramer, which was formed from purified mNeon covalent dimers (**Supplementary Fig. 8**).

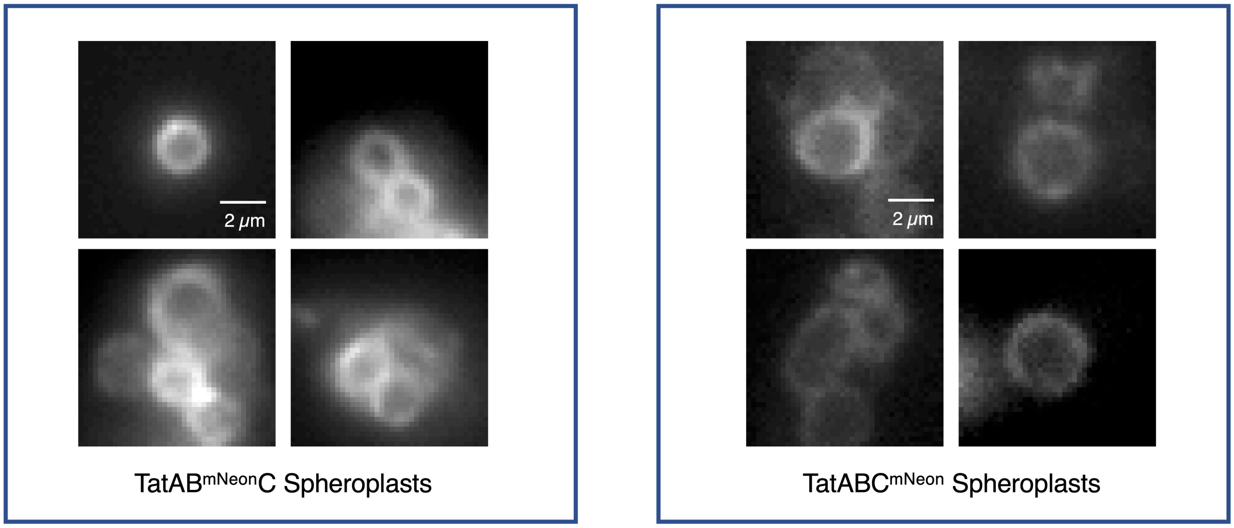

Supplementary Figure 14. Distribution of TatB^{mNeon} and TatC^{mNeon} in Spheroplast Membranes. Bacterial cells were first converted into spheroplasts before they were passed through a French press to make IMVs[7]. These images show the distribution of TatB^{mNeon} and TatC^{mNeon} within spheroplast membranes.

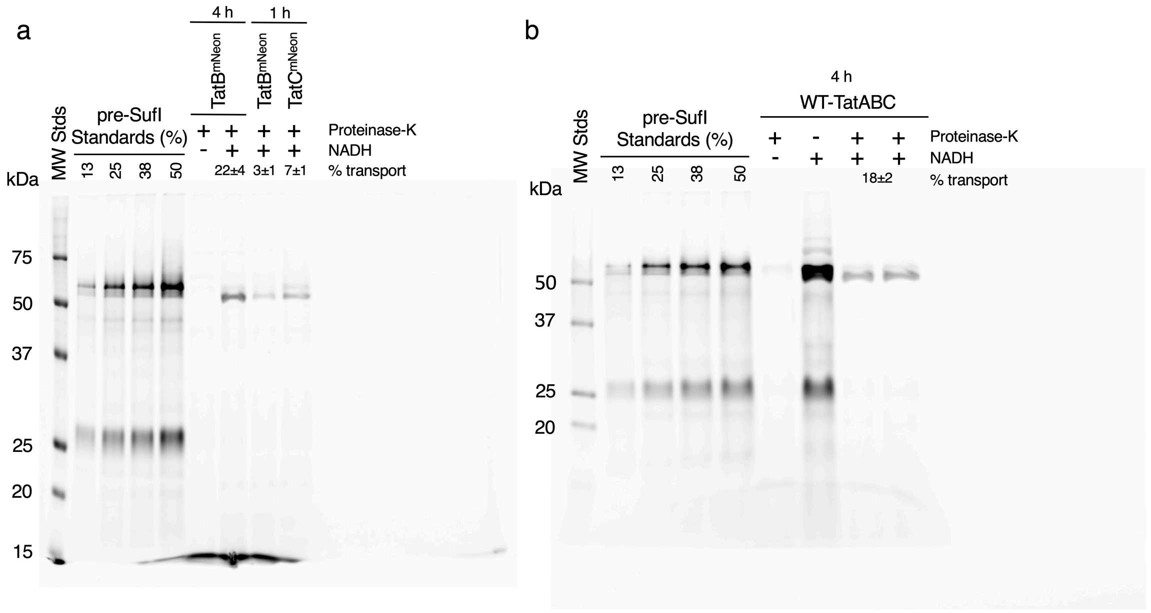

Supplementary Figure 15. Uncropped Gel Images used to create Figure 2.

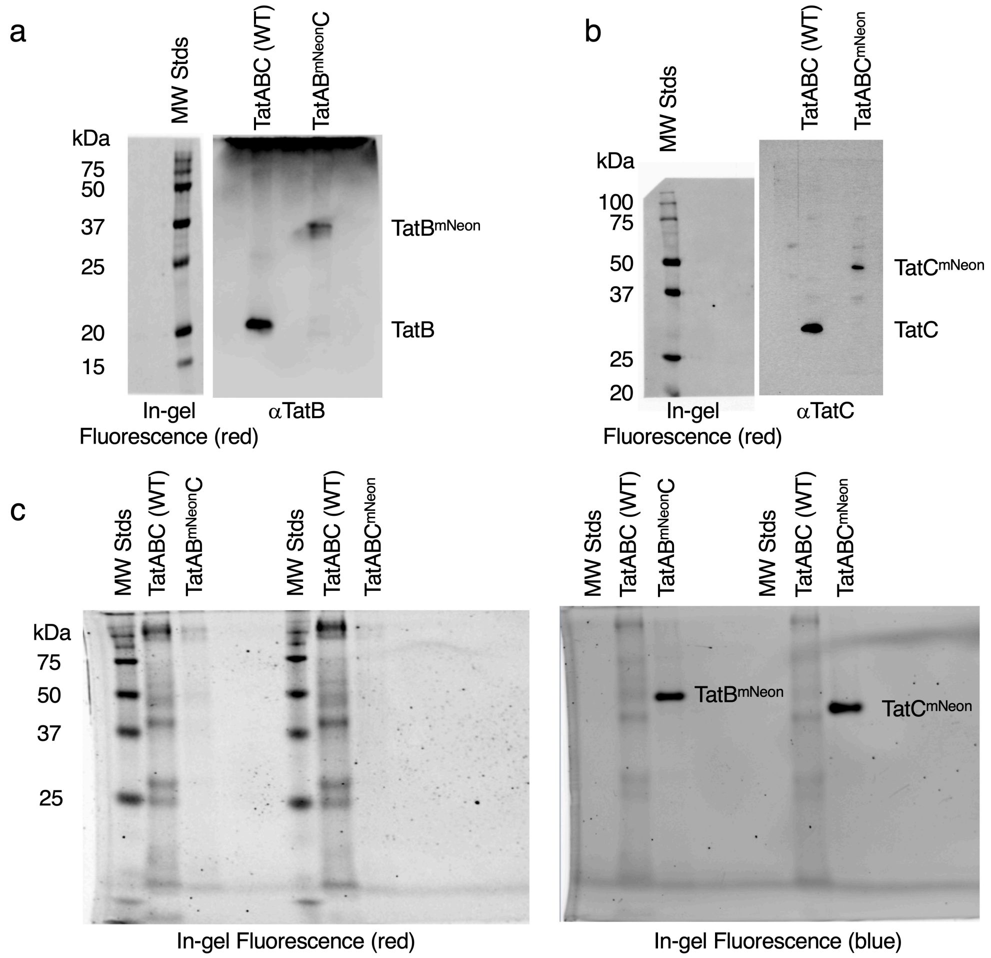

Supplementary Figure 16. Uncropped Gel and Blot Images used to create Supplementary Figure 2. "Blue" indicated excitation at 460-490 nm, and "red" indicates excitation at 650-675 nm.

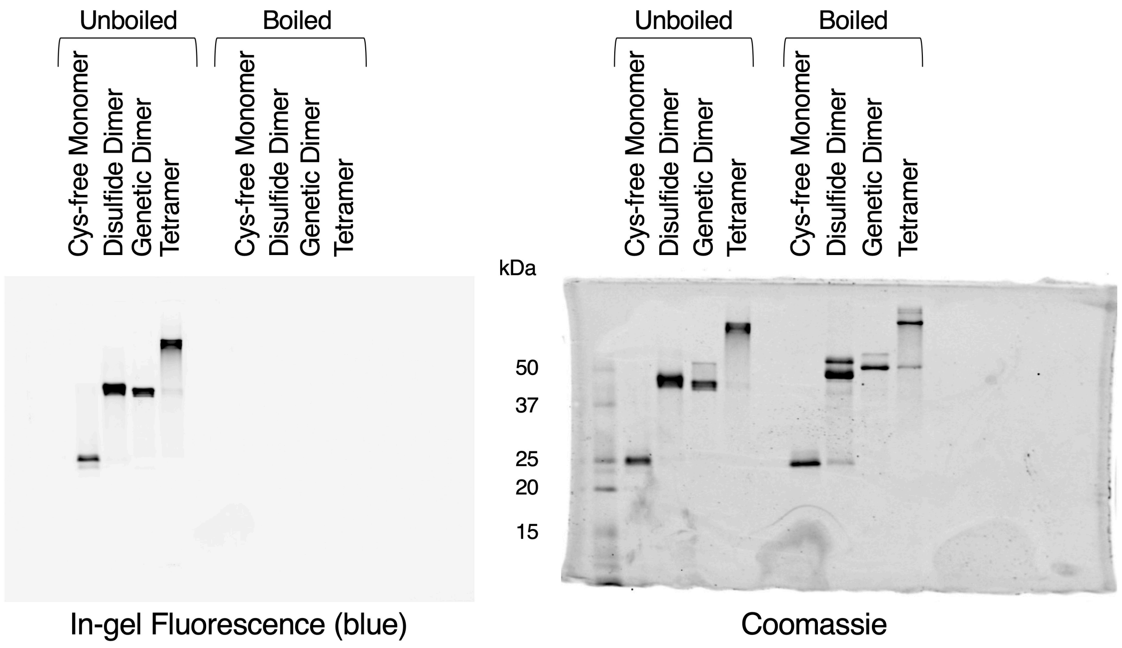

Supplementary Figure 17. Uncropped Gel and Blot Images used to create Supplementary Figure 8. "Blue" indicated excitation at 460-490 nm**.**
