# Supplementary material for: Oligomerization state of the functional bacterial twin-arginine translocation (Tat) receptor complex
Source: Commun Biol. 2022 Sep 19;5:988. doi: 10.1038/s42003-022-03952-2 (PMC9485244; doi:10.1038/s42003-022-03952-2)
Supplement: Supplementary file 3 — Description of Additional Supplementary Files [file 42003_2022_3952_MOESM3_ESM.docx]

**Description of Additional Supplementary Files**

**File name:** Supplementary Data 1
**Description:** The source data behind the graphs in the paper.

**File name:** Supplementary Software 1
**Description:** MS Excel file used to calculate the expected number of photobleaching steps for Tat complexes in IMVs.

**File name:** Supplementary Software 2
**Description:** MS Excel file used to calculate the expected number of photobleaching steps for protein oligomer standards.

**File name:** Supplementary Software 3
**Description:** MS Excel file used to calculate the standard deviation for the expectation values determined by Supplementary Software File 1. Requires the RiskAMP Plugin to run.

**File name:** Supplementary Software 4
**Description:** MS Excel file used to calculate the photon frequency histograms in Supplementary Figure 9b.
