# Supplementary material for: Oligomerization state of the functional bacterial twin-arginine translocation (Tat) receptor complex
Source: Commun Biol. 2022 Sep 19;5:988. doi: 10.1038/s42003-022-03952-2 (PMC9485244; doi:10.1038/s42003-022-03952-2)
Supplement: Supplementary file 6 — Reporting Summary [file 42003_2022_3952_MOESM6_ESM.pdf]

nature portfolio

Corresponding author(s): Siegfried Musser
Last updated by author(s): June 27, 2022

Reporting Summary

Nature Portfolio wishes to improve the reproducibility of the work that we publish. This form provides structure for consistency and transparency in reporting. For further information on Nature Portfolio policies, see our Editorial Policies and the Editorial Policy Checklist.

Statistics

For all statistical analyses, confirm that the following items are present in the figure legend, table legend, main text, or Methods section.

n/a	Confirmed	
☐	☒	The exact sample size (n) for each experimental group/condition, given as a discrete number and unit of measurement
☐	☒	A statement on whether measurements were taken from distinct samples or whether the same sample was measured repeatedly
☒	☐	The statistical test(s) used AND whether they are one- or two-sided<br>*Only common tests should be described solely by name; describe more complex techniques in the Methods section.*
☒	☐	A description of all covariates tested
☐	☒	A description of any assumptions or corrections, such as tests of normality and adjustment for multiple comparisons
☐	☒	A full description of the statistical parameters including central tendency (e.g. means) or other basic estimates (e.g. regression coefficient) AND variation (e.g. standard deviation) or associated estimates of uncertainty (e.g. confidence intervals)
☒	☐	For null hypothesis testing, the test statistic (e.g. F, t, r) with confidence intervals, effect sizes, degrees of freedom and P value noted<br>*Give P values as exact values whenever suitable.*
☒	☐	For Bayesian analysis, information on the choice of priors and Markov chain Monte Carlo settings
☒	☐	For hierarchical and complex designs, identification of the appropriate level for tests and full reporting of outcomes
☒	☐	Estimates of effect sizes (e.g. Cohen's d, Pearson's r), indicating how they were calculated

Our web collection on statistics for biologists contains articles on many of the points above.

Software and code

Policy information about availability of computer code

Data collection	Data collection was performed on a Nikon Eclipse Ti wide-field microscope as detailed in the manuscript using:<br>NIS-Elements AR 5.02.01
Data analysis	The following software packages were used for visualization, analysis, data presentation, data recording/storage, and simulations:<br>ImageJ (Fiji 1.52P)<br>Matlab R2018a<br>OriginPro 2017<br>Microsoft Excel 16.16.20 (200307)<br>SnapGene 5.3.2

For manuscripts utilizing custom algorithms or software that are central to the research but not yet described in published literature, software must be made available to editors and reviewers. We strongly encourage code deposition in a community repository (e.g. GitHub). See the Nature Portfolio guidelines for submitting code & software for further information.

Data

Policy information about availability of data

All manuscripts must include a data availability statement. This statement should provide the following information, where applicable:

- Accession codes, unique identifiers, or web links for publicly available datasets
- A description of any restrictions on data availability
- For clinical datasets or third party data, please ensure that the statement adheres to our policy

Plasmids pET-28a_mScarlet, pH6-mNeon, pH6-mNeonC, pH6-2xmNeon, pH6-2xmNeonC, pBAD_TatABmCherryC, pTatABmNeonC, pTatAkBxCs, and pTatABCmNeon

are available from Addgene (IDs# 178460-178465, 178543-45). The authors declare that all other data supporting the findings of this study are available within the paper and its supplementary information files. Raw data and materials are available from the corresponding author upon reasonable request.

Field-specific reporting

Please select the one below that is the best fit for your research. If you are not sure, read the appropriate sections before making your selection.

☒ Life sciences ☐ Behavioural & social sciences ☐ Ecological, evolutionary & environmental sciences

For a reference copy of the document with all sections, see nature.com/documents/nr-reporting-summary-flat.pdf

Life sciences study design

All studies must disclose on these points even when the disclosure is negative.

Sample size	Sample sizes were not pre-determined. Photobleaching data were compared with Monte Carlo simulations of similar dataset sizes to estimate errors.
Data exclusions	Photobleaching curves with excessive blinking were excluded. Traces with > 8 photobleaching steps were not included in the analysis as their interpretation was deemed unreliable.
Replication	Photobleaching studies on both TatBmNeon and TatCmNeon IMVs were repeated with two independent IMV preparations (results compared in Supplementary Table 1). A photobleaching dataset for the mNeonGreen standards (control) was always obtained on the same day as IMV photobleaching datasets.
Randomization	This is not relevant to this study. Single molecule fluorescence spots appeared and photobleached randomly by design. All data were analyzed as a whole, and not separated into comparison groups. Data analysis took into account this randomness by comparing to Monte Carlo simulations of the datasets.
Blinding	Data were independently analyzed by at least two individuals at various stages of data analysis.

Reporting for specific materials, systems and methods

We require information from authors about some types of materials, experimental systems and methods used in many studies. Here, indicate whether each material, system or method listed is relevant to your study. If you are not sure if a list item applies to your research, read the appropriate section before selecting a response.

Materials & experimental systems

n/a	Involved in the study	
☐	☒	Antibodies
☒	☐	Eukaryotic cell lines
☒	☐	Palaeontology and archaeology
☒	☐	Animals and other organisms
☒	☐	Human research participants
☒	☐	Clinical data
☒	☐	Dual use research of concern

Methods

n/a	Involved in the study	
☒	☐	ChIP-seq
☒	☐	Flow cytometry
☒	☐	MRI-based neuroimaging

Antibodies

Antibodies used	The anti-TatB and anti-TatC antibodies were a generous gift from T.L. Yahr and were used at the dilutions indicated in the manuscript.
Validation	The anti-TatB and anti-TatC antibodies were previously validated in Bageshwar & Musser (2007) 179:87-99.
